# Prohibitins Are Required for Cancer Cell Proliferation and Adhesion

**DOI:** 10.1371/journal.pone.0012735

**Published:** 2010-09-14

**Authors:** Claudia Sievers, Gwendolyn Billig, Kathleen Gottschalk, Thomas Rudel

**Affiliations:** 1 Research Group for Molecular Infection and Cancer Biology, Department of Molecular Biology, Max-Planck Institute for Infection Biology, Berlin, Germany; 2 Department of Biochemistry, Free University Berlin, Berlin, Germany; 3 Biocenter, University of Würzburg, Würzburg, Germany; Health Canada, Canada

## Abstract

Prohibitin 1 (PHB1) is a highly conserved protein that together with its homologue prohibitin 2 (PHB2) mainly localizes to the inner mitochondrial membrane. Although it was originally identified by its ability to inhibit G1/S progression in human fibroblasts, its role as tumor suppressor is debated. To determine the function of prohibitins in maintaining cell homeostasis, we generated cancer cell lines expressing prohibitin-directed shRNAs. We show that prohibitin proteins are necessary for the proliferation of cancer cells. Down-regulation of prohibitin expression drastically reduced the rate of cell division. Furthermore, mitochondrial morphology was not affected, but loss of prohibitins did lead to the degradation of the fusion protein OPA1 and, in certain cancer cell lines, to a reduced capability to exhibit anchorage-independent growth. These cancer cells also exhibited reduced adhesion to the extracellular matrix. Taken together, these observations suggest prohibitins play a crucial role in adhesion processes in the cell and thereby sustaining cancer cell propagation and survival.

## Introduction

Prohibitins (PHB1 and PHB2) are highly homologous proteins that are conserved throughout evolution and ubiquitously expressed [1]–[3]. Prohibitins mainly localize to the mitochondria but are also found in the nucleus and in lipid rafts of the cytoplasmic membrane [1], [4]–[7]. Localization to lipid rafts is also a feature of other members of the stomatin/prohibitin/flotilin/HflK/C-domain (SPFH) family of proteins [8].

Studies to date have associated PHB1 with diverse roles in the maintenance of cellular homeostasis. Initial work suggested it functions as an inhibitor of DNA synthesis, a role which was later assigned to its 3′UTR [9], [10]. A T-allelic expression form of PHB1 was associated with an increased risk of breast cancer [11]; however this association was not confirmed in a clinical setting [12], [13]. PHB1 co-localizes with transcription factors of the E2F family in the nucleus of breast carcinoma cells [14], and interacts with the Rb tumor suppressor protein pRB in the nucleus resulting in inhibition of the cell cycle [15]. Correspondingly, siRNA-mediated silencing of PHB1 expression was found to increase breast cancer cell proliferation [16]. Despite these indications of a tumor suppressor activity, recent studies have also demonstrated reduced cell proliferation upon loss of PHB1 expression in mouse embryonic fibroblasts and other primary cells; and the rescue of cell proliferation upon overexpression of mitochondrial-targeted PHB2 [17].

Our recent work indicates PHB1 also plays a role in cell migration: Transient silencing of PHB1 by siRNA-mediated knockdown led to a clumping phenotype in HeLa cells, comparable to that of a Her2/ErbB2 or Rac defect, i.e. clumped cells showed marked membrane staining for pan-cadherin and β-catenin. The authors suggested contact between cells were disrupted in tumor cells due to the loss of PHB1 [6]. Studies in yeast have consistently shown that PHB1 and PHB2 act as mitochondrial chaperones in the inner mitochondrial membrane [3], [18]–[20]. PHB1 and PHB2 are interdependent on the protein level and loss of one simultaneously leads to the loss of the other [21]. They interact with mitochondrial proteases, particularly mAAA, which is required in the assembly of respiratory chain complexes [22], [23]. A knock-out of prohibitins in yeast led to a reduced replicative lifespan and a defect in mitochondrial membrane potential [18], [24]. Several recent studies have also addressed the mitochondrial function of prohibitins in human cell lines: Overexpression of PHB1 was found to protect against oxidative stress [25]; in addition, siRNA-mediated knockdown of PHB2 led to the degradation of the mitochondrial fusion protein OPA1 [17] and fragmentation of the mitochondrial network [21]. However, it is still not clear whether prohibitin expression stabilizes the mitochondrial membrane potential (MMP) [17], [26], [27].

Here we show prohibitins play a role in maintaining cellular homoeostasis and proliferation in cancer cells and primary cells. Depletion of prohibitins had no major effect on mitochondrial function but did affect adhesion of cells to the extracellular matrix.

## Materials and Methods

### Cell culture

HEK293 and HeLa cells were obtained from German Collection of Microorganisms and Cell Cultures (DSMZ). HT1080wt, Jurkat and CapanI cells were obtained from American Type Culture Collection (ATCC). IF6 cells were a gift from U. Rapp, Würzburg. HeLa cells were tested and authenticated by DSMZ via STR-fingerprinting. HEK293 cells and HT1080wt cells were cultured in DMEM with 10% FCS, 1% Penicillin/Streptomycin, 10 mM HEPES pH 7.4 and 4 mM L-Glutamine. HeLa cells, IF6 cells and Jurkat cells were cultured in RPMI with 10% FCS, 1% Penicillin/Streptomycin. HT29 cells were cultured in McCoy's medium, supplemented with 10% FCS and 1% Penicillin/Streptomycin. Capan I cells were cultured in RPMI with 20% FCS and 1% Penicillin/Streptomycin. All cells were cultured at 37°C with 5% CO2.

### Generation of shRNA knockdown cell lines

shRNA-expressing vectors were constructed by cloning different shRNAs into the pLL3.7 or pLVTHM vector. To generate an inducible vector system, the shRNAs from the constitutive expression system pLVTHM were subcloned into the inducible vector system pLVPT, by transferring the cassette containing the shRNA and the H1 promoter via the XhoI/XbaI restriction sites. All constructs were verified by sequencing. Nucleotide sequences of shRNA targets used are as follows: shLuci, 5′-AACTTACGCTGAGTACTTCGA-3′; PHB1-0, 5′- GCGGAGAGAGCCAGATTTG-3′; PHB1-3, 5′-GACACATCTGACCTTCGGGAA-3′; and PHB2-0, 5′-GACAGAGAGGGCCAAGGAC-3′. Stably transduced, constitutive or inducible knockdown cell lines were constructed as described previously [28]; see also http://tronolab.epfl.ch/). Briefly, HeLa cells were transduced with the respective lentiviral vector in the presence of Polybrene (Sigma-Aldrich, St Louis, MO, USA). For the transduction of pLVPT lentiviral vectors, shRNA expression was induced by treatment with 1 µg/ml doxycycline (Sigma) for 3 days and cells were sorted for eGFP expression with the MoFlo® Flow Cytometer (Dako Denmark A/S, Glostrup, Denmark). For validation of the knockdown levels, RNA was isolated using the RNAeasy Kit (Qiagen, Hilden, Germany). Quantitative real-time PCR was performed with the QuantiTect SYBR Green real-time PCR Kit (Qiagen) according to the manufacturer's protocol. All vectors were obtained from D. Trono, Ecole Polytechnique Fédérale de Lausanne, France.

### Soft agar assay

Cells (2×10^3^) were inoculated into 0.3% low melting temperature agarose (Sigma) in DMEM supplemented with 10% FCS, and colonies were counted after 2–4 weeks of incubation at 37°C and 5% CO_2_.

### Cell proliferation and cell cycle analysis

For CFSE staining, cells were harvested and washed two times in PBS, then stained with 5 µM CFSE for 5 min at room temperature (RT). Residual CFSE was removed by washing twice with PBS and cells were seeded in 6-well plates and grown in cell culture medium. The CFSE fluorescence intensity was measured by FACS analysis every 24 hours for 5 days. BrdU assays were performed using the *Cell Proliferation ELISA, BrdU (chemiluminescent)* kit (Roche) according to the manufacturer's instructions. Briefly, cells were seeded at 3.5×10^3^ in a white rimmed 96-well plate. BrdU was added to a final concentration of 10 µM for 1 hour. Peroxidase conjugated anti-BrdU antibody was added to cells for 30 to 60 min at RT and luminescence was measured after an incubation of 3 to 10 min using a Monolight 3096 microplate luminometer (BD Bioscience).

### TMRE staining

To analyze mitochondrial membrane potential, 100 nM TMRE was added to culture medium and cells were further incubated for 30 min under normal culture conditions. Incorporation was measured by FACS. To control for the complete loss of membrane potential, 1 µM CCCP was added for 5 min.

### ATP levels

Cellular ATP synthesis was measured using an ATP *Bioluminescence Assay Kit HSII* (ROCHE), according to the manufacturer's instructions. Briefly, 10^5^ to 10^7^ cells/25 µl per well of a 96 well plate were lysed in the same volume of cell lysis reagent by incubation for 5 min at RT. Fifty microlitres of the reconstituted luciferase reagent was added to the well by automated injection (BD Bioscience, Monolight luminometer). Measurements (duration of 2 seconds) were taken after a 1 second delay.

### Western blotting

Cells were lysed in 2 x sample buffer containing 200 mM DTT and proteins were separated using 10% SDS-PAGE. After electrophoresis, the proteins were electrotransferred onto a polyvinylidene fluoride microporous membrane and immunodetected using antibodies against OPA1 (BD Biosciences), PHB1 and PHB2 (NeoMarkers) and Actin (Sigma). OPA1 fragmentation was quantified using AIDA software.

### Immunofluorescence staining

Cells grown on coverslips were fixed with 4% paraformadehyde for 30 min at RT, and then permeabilized in 0.5% Triton X-100. Next, cells were immunostained using the following antibodies: mouse anti-Tom20 (BD Biosciences) (1∶100 dilution, 2 h RT), mouse anti-Paxillin-1 (BD Transduction) (1∶100), phalloidin-alexa 488 (MoBiTec) (1∶200) and donkey anti-mouse, Cy™3 (1∶100). Quantification of the mitochondrial network was done using ImageJ software.

### Transmission electron microscopy

Cells depleted of prohibitins by inducing shRNA expression for 10 days were fixed with 2.5% glutaraldehyde, postfixed with 0.5% osmium tetroxide and contrasted using tannic acid and uranyl acetate. Specimens were dehydrated in a graded ethanol series and embedded in Polybed. Ultrathin sections were analysed in a Leo 906E transmission electron microscope (Leo GmbH).

### Adhesion assay

To measure adhesion of cells, microplates were coated with 1 mg/ml fibronectin, 2 mg/ml collagen or 3 mg/ml BSA, respectively. GFP expression was below the sensitivity of the fluorometer (at 488 nm), therefore we stained cells with CFSE prior to seeding. Cells were incubated on coated wells for 30 min at 37°C.

To analyze cellular migration and adhesion, cells induced with doxycycline for 8 days were seeded under subconfluent conditions, in cell culture plates or coverslips, for 20 h followed by 4 h serum starvation. The cells were treated with 50 µM Forskolin for 20 min to induce cAMP/PKA activation and thereby lamellipodia and focal adhesion formation. Actin filaments and focal adhesions were visualized via immunocytochemistry and focal adhesions were counted manually.

## Results

### Selective loss of PHB1 depleted cells

**Figure 1:**
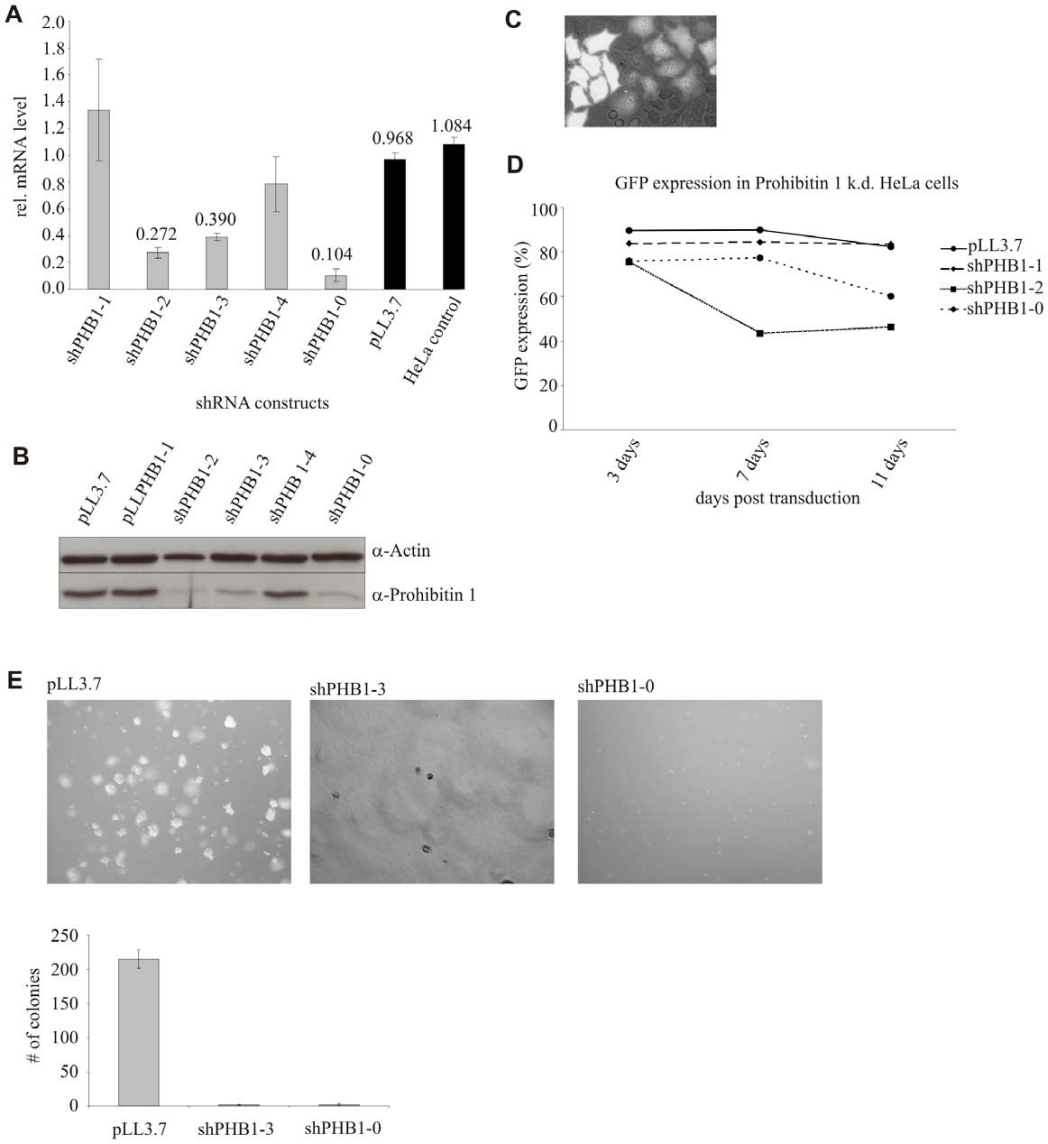
Silencing of PHB1 led to a decrease in cell proliferation and anchorage-dependent growth in HeLa cells. (A, B) PHB1 mRNA and protein levels were efficiently reduced in cells expressing shPHB1-0, shPHB1-2 and shPHB1-3 using the constitutive lentiviral expression system pLL3.7 as was shown by qRT-PCR and Western Blots, respectively. (C) Upon lentiviral transduction stable integration is indicated by GFP expression. (D) With the expression of positively validated shRNAs targeting PHB1 mRNA the fraction of GFP expressing cells was reduced within ten days of transduction from a pool of transduced cells within, as shown by FACS analysis. (E) Transduced HeLa cells expressing control vector or PHB1 targeting shRNAs were seeded in soft agar. Only control cells grew under anchorage-independent conditions and formed colonies.

To date no consensus on the function of PHB1 has been reached; in breast cancer cells, PHB1 has been reported to act as a tumor suppressor [15], [16], whereas in primary endothelial cells depletion of PHB1 resulted in reduced cell proliferation [26]. To define the role of PHB1 in HeLa cells, an adenocarcinoma cell line, we generated stable cell lines expressing PHB1-directed shRNAs using the constitutive pLL3.7 lentiviral shRNA expression system [29]. Expression of three different shRNAs, shPHB1-2, shPHB1-3 and shPHB1-0, targeting the coding region of PHB1 induced the depletion of PHB1 by RNA interference (RNAi) at the mRNA (Fig. 1A) and protein (Fig. 1B) level. The depletion of PHB1 correlated with the simultaneous expression of the marker protein eGFP (Fig. 1C), indicating the successful lentiviral transduction of these cells. We then grew the different cell lines and monitored the composition of the cell population using microscopy and FACS analysis. A selective reduction of shPHB-expressing cells from the cell pool was observed, visible by a reduction in the eGFP-positive cell population (Fig. 1D). This loss was not due to increased apoptosis of the shRNA-expressing cells (data not shown) but faster growth of non-transduced, PHB1 protein-expressing cells, and therefore eGFP-negative cells, which showed normal proliferation.

To test whether this observation was specific for HeLa cells, a number of different cancer cell lines, including Capan1 cells, HT1080wt, Jurkat T cells, IF6 and HT29 cells, were transduced with the same lentiviral shRNA construct and their growth monitored as before. Western blot analysis revealed expression of shPHB1-0 resulted in depletion of PHB1 in all cell lines tested (Fig. S1A). In addition, similar to HeLa cells, prolonged cultivation resulted in a loss of eGFP-positive cells (Fig. S1B), ruling out a cell line-specific effect. Interestingly, a similar phenomenon was observed in proliferating primary human umbilical vein endothelial cells (HUVEC) after transducing shPHB1-0 (data not shown), suggesting that loss of PHB1 interferes with proliferation.

Since anchorage-independent growth is a hallmark of cancer cells [30], we also assessed this phenotype in our cell lines constitutively expressing shRNAs targeting PHB1. Cells were seeded in soft agar and their growth monitored as before. Control and non-transduced cells exhibited normal growth characteristics, forming colonies and proliferating, whereas PHB1-knockdown cells remained as single cells (Fig. 1E), indicating PHB1 plays a role in anchorage-independent growth.

### PHB1/PHB2 are required for proliferation of cancer cells

Since conditional silencing of PHB1 expression led to loss of proliferation and consequently an overgrowth by untransduced cells, long-term studies on the effects of reduced PHB1 expression at the molecular level were not possible. Therefore, we expressed the same validated shRNAs for the functional knockdown of PHB1 in a lentiviral vector under the control of a doxycycline-inducible promoter [28]. To analyze the role of PHB2 and its interaction with PHB1, we designed an additional shRNA specifically targeting PHB2 mRNA (for details see Materials and Methods).

**Figure 2:**
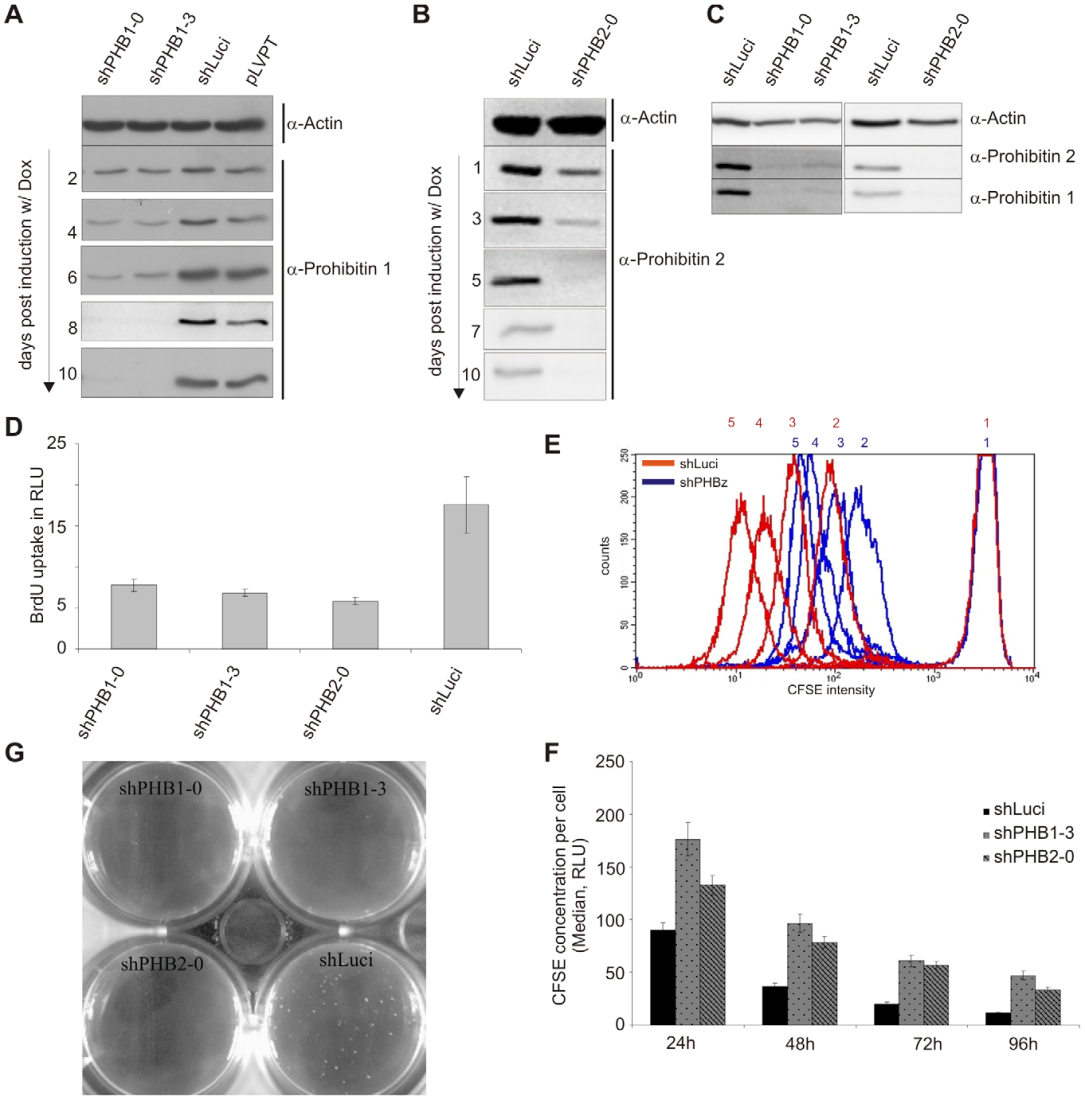
Inducible expression of shRNAs targeting either PHB1 or PHB2 led to depletion of both proteins and subsequently to a slower cell proliferation rate. (A, B) Transduction with the pLVPT lentiviral system allows doxycycline inducible shRNA expression. Within the indicated time of induction PHB1 and PHB2 protein expression was efficiently reduced. (C) The inducible expression of either PHB1 targeted or PHB2 targeted mRNA led to a reduced expression of both proteins, as shown by Western blot. (D) BrdU incorporation analysis shows HeLa cells expressing shPHB1-0, shPHB1-3 or shPHB2-0 for 10 days exhibit a reduced proliferation rate in comparison to cells expressing control shRNA (shLuci). (E) CFSE intensity as measured by FACS at 0 h (1), 24 h (2), 48 h (3), 72 h (4), and 96 h (5) is higher in prohibitin knockdown cells indicating reduced dilution of CFSE to fast replicating daughter cells. (F) CFSE intensity was gauged by FACS every 24 h for 5 days and the median of each peak was plotted against each other. (G) Inducible expression of shRNAs resulted in the same phenotype of reduced/loss of anchorage-independent growth in PHB1 (shPHB1-0, shPHB1-3) and PHB2 (shPHB2-0) knockdown cells as observed with the constitutive expression system.

Reductions in PHB1 and PHB2 protein expression were observed 3 to 4 days after the addition of doxycycline, becoming markedly reduced at 5–6 days and then remaining stably reduced for up to 10 days (Fig. 2A, B). Depletion of one prohibitin protein led to the loss of the other (Fig. 2C). This effect was probably due to protein destabilization as mRNA levels of the non-silenced gene remained constant (data not shown). As previously observed upon constitutive silencing of PHB1 (Fig. 1D), inducible silencing of prohibitins led to reduced proliferation of cells. BrdU incorporation assays revealed reduced DNA synthesis 5 days post induction of PHB1 and PHB2 depletion (Fig. 2D). To assess the proliferation rate at a single-cell level, cells were stained with Carboxy fluorescent succinidylester (CFSE); this stain is evenly distributed in the cytoplasm and stoichiometrically distributed to daughter cells during cell division. Cell proliferation was clearly decreased in PHB1- or PHB2-depleted cells in comparison to control cells (Fig. 2E). Furthermore, these cells showed reduced anchorage independence and failed to grow on soft agar (Fig. 2F). Thus, our data demonstrates that prohibitins play a role in cell proliferation.

### Loss of prohibitins leads to fragmentation of mitochondria

**Figure 3:**
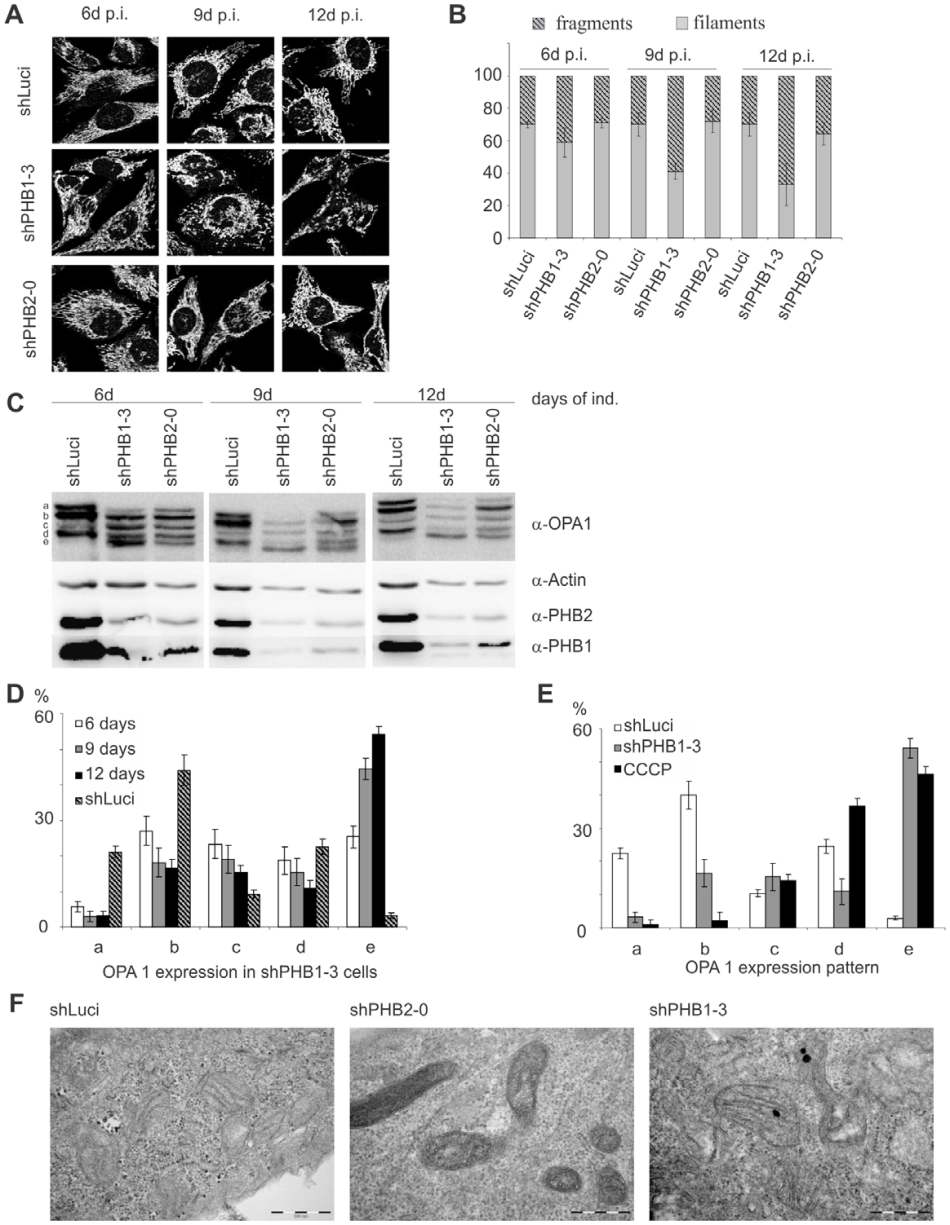
Prohibitin knockdown led to fragmentation of the mitochondrial network without affecting cristae morphology. (A, B) Mitochondria from prohibitin knockdown cells were stained with anti-Tom 20 and mitochondrial fragmentation was quantified using ImageJ software. An extended induction with doxycycline led to an increased fragmentation in shPHB1-3 expressing cells. (C) Western blots showing that prolonged doxycycline-induced shPHB1-3 expression led to a stronger prohibitin knockdown than shPHB2-0 expression, correlating with an increasing fragmentation of the fusion competent fragments a and b of OPA1. (D) Quantification showed that the fusion competent forms were degraded into the smaller fragments c and e. (E) Quantification of OPA1 patterning in control cells (shLuci), PHB1-depleted cells (shPHB1-3) and cells treated with 60 µM CCCP for 80 min (CCCP) to induce loss of ΔΨm. The OPA1 pattern in CCCP-treated cells differed from shPHB1-3 expressing cells, as the fragments a and b were completely lost while the smaller fragments d and e were increased. (F) TEM pictures of cells with a prohibitin knockdown induced for 10 days showed a more dense appearance of mitochondria than shLuci-expressing control cells, but the morphology of the cristae structure was not changed.

Since prohibitins are mainly localized to mitochondria [1], we decided to test whether depletion of prohibitins affected the integrity of mitochondria. Using immunofluorescence microscopy we observed a clear fragmentation of the mitochondrial network in cells expressing shPHB1-3, which increased over time (Fig. 3A,B); Expression of shPHB2-0 failed to induce a significant increase in fragmentation of the mitochondrial network (Fig. 3A,B). In addition, expression of shPHB1-3 resulted in a more efficient knockdown of both prohibitins.

Amongst many other proteins, OPA1 plays a major role in the control of mitochondrial fusion [31] and cristae integrity [32]. Since previous publications have shown that prohibitins are necessary for stable expression of OPA1 [17], [21], we analyzed the effect of PHB1 and PHB2 depletion on the levels of mitochondrial fusion protein OPA1. OPA1 proteins are expressed as seven splice variants, visible as five bands (a–e) on a Western blot, attributed in part to the fusion activity of OPA1 [33]. We observed degradation of OPA1 in cells depleted of prohibitins, which correlated with the efficiency of prohibitins depletion. In particular, the fusion competent fragments a and b were lost and the small fragments c–e increased upon PHB1 depletion for longer time periods (Fig. 3C, D). Interestingly, in cells expressing shPHB2-0 bands a and b remained intact, and only increases in band e were observed (Fig Fig. 3C). The effect was specific for OPA1 since degradation of other mitochondrial protein, including Hsp60, ATP synthase and Tims and Toms was not observed (data not shown). These results support a selective activity of the shPHB1-3 in mitochondrial fragmentation and OPA1 degradation.

From previous studies it was known that dissipation of the MMP induces OPA1 degradation and mitochondrial fragmentation [33]. Therefore, we tested whether the pattern of OPA1 fragmentation induced by PHB1 depletion and MMP was similar. Cells were treated with the uncoupling reagent carbonyl cyanide *m*-chlorophenyl hydrazone (CCCP) and the OPA1 bands were detected by immunoblot. OPA1 protein patterns were clearly different in cells without MMP in comparison to those depleted of PHB1 (Fig. 3E). The fusion competent fragments of OPA1 were degraded and expression of fragments c, d and e was increased in the absence of MMP. In prohibitin-depleted cells, expression of band d expression was strongly decreased, whereas band b was only slightly decreased, suggesting that different mechanisms account for the observed effects. In a time-course experiment, we observed that CCCP causes mitochondria fragmentation prior to OPA1 degradation (data not shown), indicating that OPA1 degradation may be a consequence of mitochondrial fragmentation in this case.

To test whether silencing of PHB1 induces loss of MMP, we stained shPHB1-3 expressing cells with tetramethylrhodamine ethyl ester perchlorate (TMRE), a cell-permeable dye that is sequestered by active mitochondria. TMRE staining intensity was similar in control and PHB1-depleted cells (Fig. S2A), suggesting that the mitochondrial membrane remains intact despite fragmentation of mitochondria in these cells. In accordance, ATP levels remained unchanged in cells depleted of prohibitins (Fig. S2B). Since OPA1 degradation has also been shown to lead to changes in cristae morphology [32], we investigated the cristae structure of mitochondria in cells depleted of prohibitins using Transmission Electron Microscopy (TEM). In PHB1-silenced and control cells, cristae structure remained intact (Fig. 3F), indicative of a normal mitochondrial physiology.

### Cell- to- cell contact is inhibited in prohibitin depleted cells

**Figure 4:**
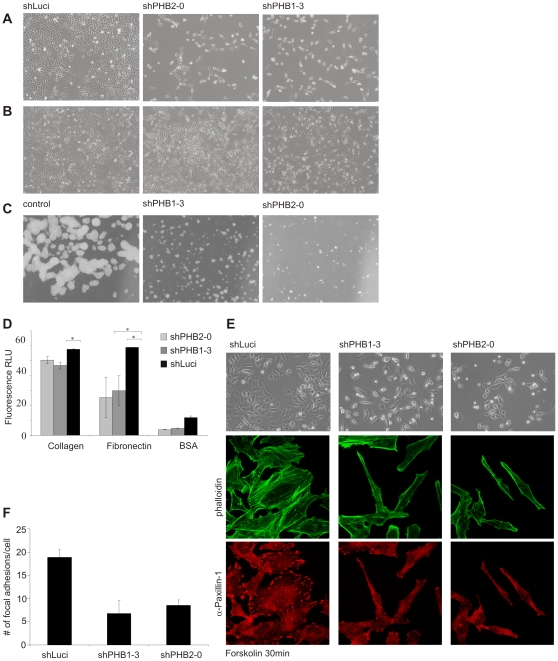
Prohibitin knockdown led to a changed morphology in HeLa cells and to reduced cell-cell contact. (A, B) Silencing prohibitin expression for 10 days resulted in a stretched cell morphology with the nucleus slightly raised. (A) When seeded at low density prohibitin knockdown cells remained as single cells with minimal cell-cell contact. (B) At higher seed density, prohibitin-knockdown cells still showed an elongated morphology but either formed clusters (shPHB1-3) or remained predominantly as single cells with reduced cell-cell contact. The proliferation rate of prohibitin knockdown cells was also increased in comparison to cells seeded at low to normal density. (C) Preventing anchorage by seeding cells on agar-coated tissue culture plates strongly reduced proliferation rate and colony formation in prohibitin knockdown cells, whereas control cells (shLuci expressing cells) formed large cell clumps. (D) Prohibitin-depleted and control cells were treated with CFSE to increase fluorescence intensity and seeded on extracellular matrix proteins collagen or fibronectin, or on BSA for 30 min. Non- adherent cells were washed off and fluorescence intensity was measured. Prohibitin knockdown significantly reduced adhesion. (E) Doxycycline-induced cells were seeded on coverslips for 20 h followed by serum starvation for 4 h. After treatment with 50 µM Forskolin for 20 min, brightfield images showed lamellipodia formation in control cells but not in prohibitin- depleted cells. Furthermore, prohibitin-depleted cells showed decreased formation of focal adhesions as shown by immunocytochemistry staining using phalloidin and anti-paxillin antibodies. (F) For the quantification of focal adhesions in control and prohibitin knockdown cells, α-paxillin 1 stained spots were counted in 30 cells per condition.

During our initial studies on the prohibitin knockdown cell lines, we observed that the loss of prohibitin-depleted cells was dependent on the density at which the cells were seeded in cell culture plates. At high cell densities, the loss of knockdown cells was reduced but not prevented (Fig. 4A). Moreover, loss of prohibitins expression caused a change in cell morphology in certain cancer cell lines: HeLa cells, especially when seeded at low density tended to remain as single cells with an elongated morphology, reminiscent of fibroblasts (Fig. 4B). A similar phenotype was observed when HeLa cells were kept under suspension conditions by seeding them on agarose-covered cell culture dishes: only control cells formed colonies, while prohibitin-silenced cells failed to form cell- to-cell contacts and colonies (Fig. 4C).

To study cell-matrix contact formation, cells were seeded onto plates coated with the extracellular matrix proteins fibronectin or collagen. Adhesion of PHB1- and PHB2-depleted cells to these matrix proteins was significantly inhibited (Fig. 4D). To further quantify cell-matrix attachment, focal adhesion formation was induced by Forskolin after serum starvation. Immunofluorescence microscopy revealed reduced spreading and formation of actin filaments (Fig. 4E), plus a reduction in the numbers of focal adhesions (Fig. 4E, F). These data suggest that prohibitins also play a role in the cell-matrix interaction.

## Discussion

Since the discovery of their anti-proliferative activity, prohibitins have been implicated in multiple cellular functions [10], [18], [34]. However, several lines of indirect evidence, such as their overexpression in numerous cancer cells [35], indicated that prohibitins were not functioning as a classical tumor suppressor. Here, we show prohibitins are a crucial prerequisite for cancer cell proliferation and survival. We used lentivirus-mediated shRNA expression to specifically reduce PHB1 and PHB2 mRNA and protein levels, respectively. Using the constitutive expression system pLL3.7, we demonstrated that, in all tested cancer cell lines, numbers of cells, expressing a PHB1 mRNA targeting shRNA were decreased from within a pool of transduced and non transduced cells. Furthermore, PHB1-depleted cells were unable to grow under anchorage-independent conditions. Our results are in accordance with two recent studies demonstrating that RNAi-mediated silencing of PHB1 or PHB2, respectively, leads to reduced proliferation in primary endothelial cells and mouse embryonic fibroblasts [17], [26]. These data are in stark contrast to a previous study showing increased proliferation in the breast cancer melanoma cell line MCF-7 upon siRNA-mediated silencing of PHB1 [16]. It is possible prohibitins play a different role in breast cancer cells in comparison to other cancer cell types.

In agreement with the initial finding that the RNA of 3′UTR of PHB1 exerts cytotoxic effects on cancer cells [9], [10], the expression of a T-allelic PHB1 3′UTR has been linked with an increased susceptibility to breast cancer [11]: however, several clinical studies have contradicted these findings [12], [13]. In addition, sequencing of the PHB1 3′UTR of different cancer cell lines, including MCF-7, revealed a higher frequency of overall sequence variation in comparison to primary non-cancer cells rather than a predominant expression of a specific T-allele (CS and TR, unpublished). These findings suggest that prohibitins do play a role in carcinogenesis, but the level at which the effect is exerted, i.e. 3′UTR RNA or protein, remains unclear: Silencing of either PHB1 or PHB2 mRNA by RNAi resulted in reduction of the respective 3′UTR but also in a knockdown of both prohibitin proteins. Since expression of all tested shRNAs led to a slowed proliferation rate, this phenotype is most likely a consequence of the loss of prohibitin proteins.

To assess the long-term effect of prohibitin depletion, we generated HeLa cell lines with inducible shRNA expression. In these cells PHB1 and PHB2 mRNAs were efficiently and selectively down-regulated upon induction of the gene-specific shRNA and prohibitin proteins were reversibly suppressed. Similar to the constitutive silencing of PHB1 or PHB2, induction of shRNA expression reduced the proliferation of cells and prevented colony formation on soft agar plates, indicative of a defect in anchorage-independent growth. These cells lines enabled us to fine-tune PHB1 and PHB2 silencing under standardized conditions and to investigate the mechanism underlying the strong phenotype associated with prohibitin depletion. We first analyzed the effect of prohibitin depletion on the levels of mitochondrial fusion protein OPA1 as previous publications have shown that prohibitins are necessary for stable expression of OPA1 [17], [21]. We could show that OPA1 expression is dependent on the levels of prohibitins. A slight reduction of prohibitins, seen early after induction of shRNA production, led to a partial fragmentation of the fusion competent OPA1 fragments a and b. At later time points of induction and consequently stronger depletion of prohibitins, OPA1 fragmentation increased and mitochondria appeared fragmented. An incomplete reduction of prohibitin proteins, as seen with expression of shRNAs shPHB1-0 and shPHB2-0, only resulted in mild OPA1 fragmentation and no fragmentation of the mitochondrial network. Further analysis revealed that even strong and long-lasting depletion of PHB1 e.g. in cells producing shPHB1-3, did not cause the dissipation of MMP.

To date, the effects of prohibitin loss in mammalian cells are still unclear: Schleicher et al. [26] and Ross et al. [27] show a reduction in MMP upon PHB1 and PHB1/2 depletion via RNAi in primary endothelial cells and a T-cell line, respectively; in contrast, Merkwirth and colleagues [17] demonstrated that although PHB2 depletion in MEFs causes OPA1 fragmentation, respiratory activity and MMP are not affected. Treatment of cells with the ionophore CCCP resulted in the rapid loss of MMP accompanied by a gradual and consecutive fragmentation of the OPA1 fusion-competent protein fragment and the mitochondrial network. Different patterns of OPA1 fragmentation were observed in CCCP treated cells and prohibitin-depleted cells: All fusion incompetent fragments (c–e) were upregulated upon CCCP treatment, but not as a consequence of prohibitin reduction. In addition, fragment d was downregulated when prohibitin levels were reduced, but upregulated upon CCCP treatment. Thus, our data confirm the findings of Merkwirth at al. [17], showing downregulation of fragment d (corresponding to fragment S4) in PHB2-depleted MEFs.

Studies in yeast have shown prohibitins interact with mAAA protease and are important for the proper integration of respiratory chain complex proteins [3], [23]. Furthermore, rhomboid like protease PARL is thought to be involved in OPA1 processing [36]. Considering the fragmentation pattern of OPA1 in prohibitin knockdown cells, it seems most likely that multiple proteases are involved in OPA1 processing and not all of them are affected by prohibitin loss. This would be in accordance with our observation that both mitochondrial cristae morphology and ATP synthesis were unaffected by a loss of prohibitins.

Mitochondrial fragmentation only occurred in cell with a strong depletion of prohibtins as we saw it upon shPHB1-3 expression. However, this phenotype did not correlate with the observed proliferation defect which could already be observed in cells with a slight reduction of prohibitin levels. Here, the expression of shPHB2-0 which only resulted in a medium knockdown of prohibitins caused a severe proliferation defect suggesting that the functions of prohibitins in proliferation and mitochondrial physiology differ. What did correlate was the occurrence of the adhesion defect and the observed reduction in proliferation. For instance, upon expression of shPHB2-0, cells already showed a decrease in cell-cell contact and cell-matrix formation and were sensitive to cell density (Fig 4A). At the same time, the proliferation rate was strongly reduced in these cells.

In prohibitin depleted cells, the ability to adhere to fibronectin is impaired (Fig. 4D). Experiments with Forskolin, an activator of cAMP/PKA signaling showed that prohibitin knockdown cells also have a defect in forming lamellipodia and focal adhesions, both being critical events for cell movement and migration [37]. These findings are in accordance with our previous work showing siRNA-mediated silencing of PHB1 leads to a defect in cellular migration [6], [38]. Considering these results, the role of prohibitins in regulating cell proliferation seems to be associated with adhesion/migration signaling. Localization studies show prohibitins accumulate predominantly in mitochondria. However, both proteins belong to the SPFH domain family and are transmembrane proteins. Members of this family, e.g, Flotilin, are expressed in lipid rafts. Furthermore, we [6] and others [39] have previously isolated prohibitins from lipid rafts. It is therefore conceivable that prohibitins play an important role at the plasma membrane, most likely as chaperones and scaffolding proteins as in mitochondria.

Our findings clearly show that prohibitins are required for proliferation and growth of cancer cells and regulation of cellular homoeostasis. We could show their crucial role in cancer cell propagation and survival while implicating an important function in cell adhesion and cell contact formation. This leads to an overall perception of prohibitins as chaperoning proteins not only in the mitochondria but throughout the cell.

## Supporting Information

Figure S1 Prohibitin 1 silencing leads to a decrease in cell proliferation and anchorage dependent growth in various cancer cell lines. (A) PHB1 protein levels were efficiently reduced with expression of the shRNA shPHB1-0 in HT29, IF6 and HT1080 cells. (B) FACS analysis showed that the fraction of GFP expressing cells was reduced within nine days of transduction from a pool of transduced cells. These include various cancer cells as well as primary cells. (C) Transduced cancer cells expressing control vector or prohibitin 1 targeting shRNAs were seeded in soft agar. Only control cells grew under anchorage independent conditions and formed colonies.
(0.32 MB JPG)

Figure S2 Mitochondrial function is not impaired in prohibitin-depleted cells. (A) To analyze the MMP upon prohibitin depletion, cells were stained with 100 nM TMRE for 30 min and analyzed by FACS. To induce a loss of MMP, cells were treated with 1 µM CCCP for 5 min. (B) The MMP was not changed compared to that of control cells (shLuci). Measurement of ATP levels using the ATP Bioluminescence Assay Kit HSII (ROCHE) showed no reduction in prohibitin-depleted cells.
(0.11 MB JPG)
