# Supplementary figures and images for: Prohibitins Are Required for Cancer Cell Proliferation and Adhesion
Source: PLoS One. 2010 Sep 14;5(9):e12735. doi: 10.1371/journal.pone.0012735 (PMC2939069; doi:10.1371/journal.pone.0012735)

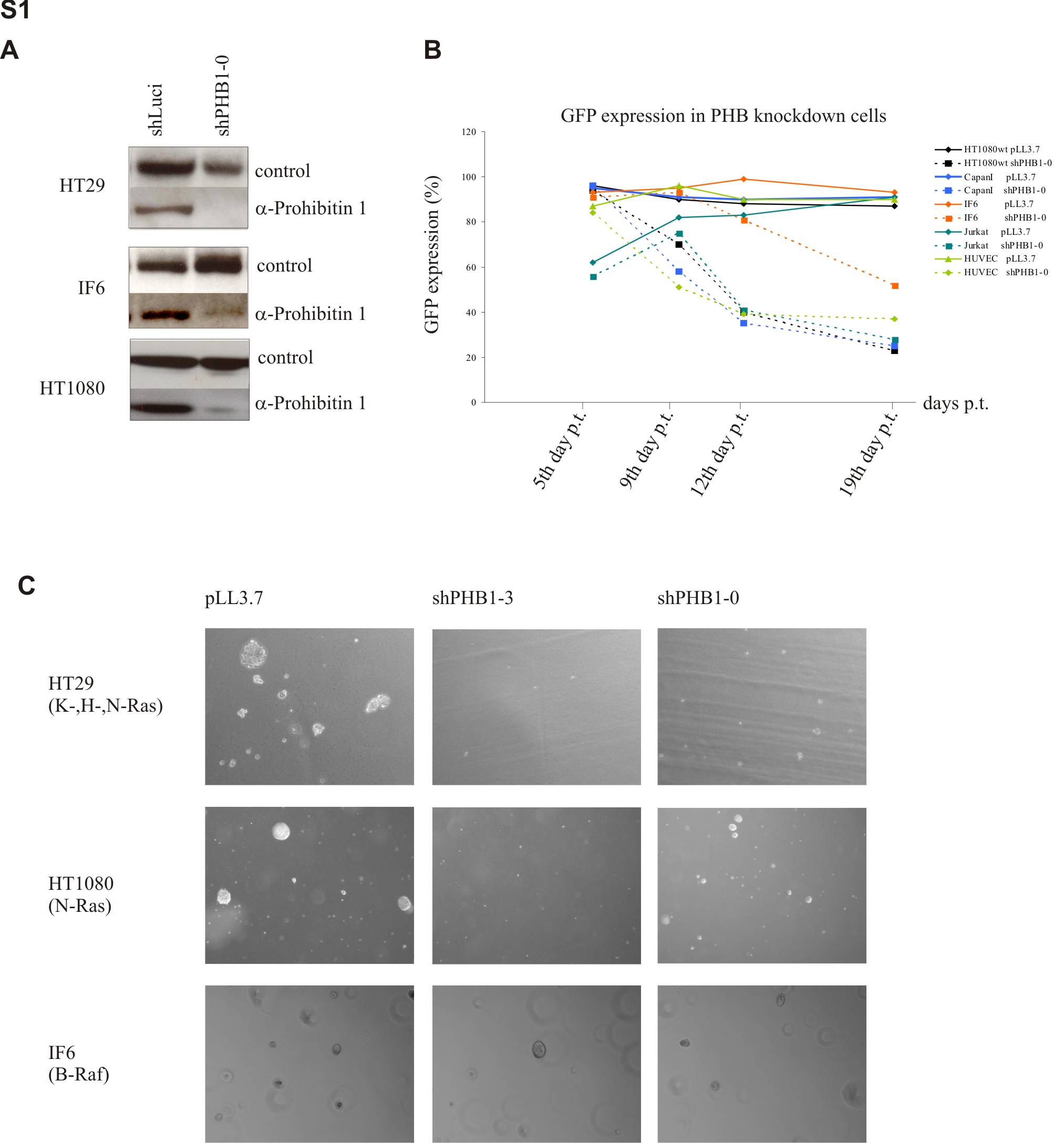

Supplement: Figure S1 — Prohibitin 1 silencing leads to a decrease in cell proliferation and anchorage dependent growth in various cancer cell lines. (A) PHB1 protein levels were efficiently reduced with expression of the shRNA shPHB1-0 in HT29, IF6 and HT1080 cells. (B) FACS analysis showed that the fraction of GFP expressing cells was reduced within nine days of transduction from a pool of transduced cells. These include various cancer cells as well as primary cells. (C) Transduced cancer cells expressing control vector or prohibitin 1 targeting shRNAs were seeded in soft agar. Only control cells grew under anchorage independent conditions and formed colonies. (0.32 MB JPG) [file pone.0012735.s001.jpg]

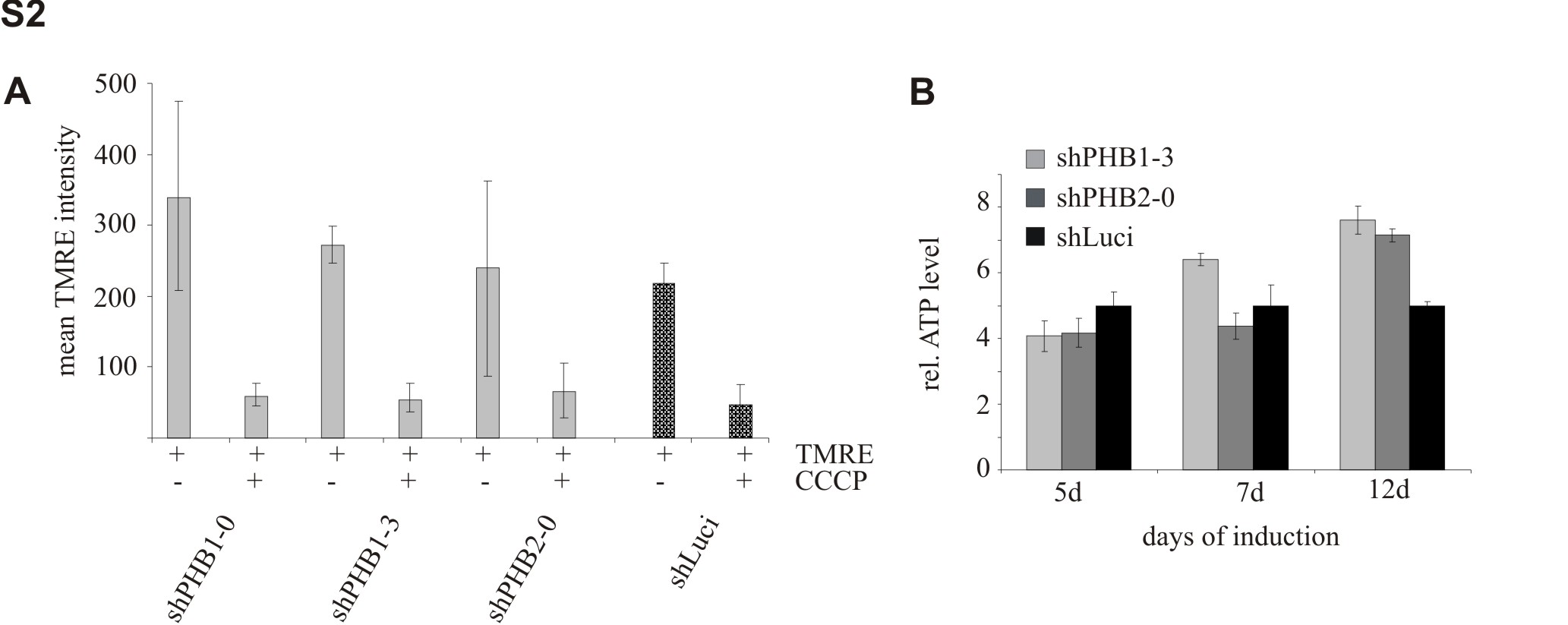

Supplement: Figure S2 — Mitochondrial function is not impaired in prohibitin-depleted cells. (A) To analyze the MMP upon prohibitin depletion, cells were stained with 100 nM TMRE for 30 min and analyzed by FACS. To induce a loss of MMP, cells were treated with 1 µM CCCP for 5 min. (B) The MMP was not changed compared to that of control cells (shLuci). Measurement of ATP levels using the ATP Bioluminescence Assay Kit HSII (ROCHE) showed no reduction in prohibitin-depleted cells. (0.11 MB JPG) [file pone.0012735.s002.jpg]
